# Effect of mixture design approach on nutritional characteristics and sensory evaluation of steamed bread added rice flour

**DOI:** 10.3389/fnut.2022.989090

**Published:** 2022-11-11

**Authors:** Shuangqi Tian, Yichun Wei, Zhicheng Chen

**Affiliations:** College of Food Science and Technology, Henan University of Technology, Zhengzhou, China

**Keywords:** mixture design approach, steamed bread, flour, rice flour, nutritional characteristics

## Abstract

This study was designed to evaluate the effects of different rice nutrient compounds on steamed bread’s nutritional characteristics and sensory evaluation. The mixture design approach was used to research the interactions between different rice flours and wheat flours on the sensory evaluation of steamed bread. The arginine content of different rice flour (long-grained rice, polished round-grained rice, and black rice) was higher at 44.19, 21.74, and 34.78% than that of the common wheat, respectively. When the added amount of mixed rice flours exceeds 15%, the steamed bread gradually reduces its elasticity, and sensory score, and has a smaller specific volume. Rice is a widely consumed grain product, which provides energy and nutrients for more than half of humanity, especially in Asia. Different rice varieties have received increased attention from researchers for their high bioactive substances and other health benefits. The results of the current study provide a theoretical basis for the nutritional steamed bread and noodle industries to use different rice flour as an ingredient for enhancing or to improving the nutritional value of flour products.

## Introduction

A significant number of researches have recently been carried out to develop gluten-free products by removal of gluten from wheat flour products, such as noodles, biscuits, cake, and steamed bread, which introduces some significant technological challenges (1–3). These challenges are indispensable for achieving desired farinograph properties, gas-holding ability, and texture characteristics of dough (4–11). Alternative compositions such as starch and protein from different flour sources have been recently used to acquire previously mentioned properties to satisfy its technological functions. However, the use of different flour sources is limited because manufacturers are still accustomed to using refined, enriched other flour sources (5, 12–14).

Rice is a widely consumed grain, which provides energy and nutrients for more than half of humans, especially with regard to Asians (15). Recently, different rice varieties have received increased attention from researchers for their high bioactive substances, resistant starch, and other health benefits (16). Rice flour is utilized in a wide variety of food products, so it can affect the final product quality (17, 18). Different rice flours have also become a popular raw material in many health food products because of consumers’ health concerns (19). It is deemed to be a more nutritious and healthier product than non-organic flours due to the wide use of heavy metal contamination, pesticide residue, herbicides, and chemical fertilizers (20). Variations in the properties of different rice flours have significant effects on the overall quality of the food products (21). Oil absorption capacities of different rice flours are associated with rancidity that naturally occurs in food products, which have a major predictor of shelf-life (12, 22, 23). The high-viscosity of different rice flours could be properly employed in high-viscosity products as a thickening agent (24).

Recently, more attention has been focused on color-grained rice varieties, which contain polyphenols in the aleurone and the pericarp, respectively, whereas common rice varieties contain small amounts of polyphenols (25–28). Polyphenols, such as phenolic acids, anthocyanins, and proanthocyanidins, have been reported as the primary antioxidants in different rice varieties (29–32). Generally, white rice contains mainly phenolic acids, and the presence of procyanidins characterizes red rice; however, black rice is characterized by the presence of anthocyanins (33–35).

However, little is known about the effect of different rice flours on steamed bread’s nutritional characteristics and performance (36–39). Therefore, it is necessary to illustrate how different color-grained rice flours contribute to steamed bread’s nutritional characteristics and performance (40). So, three different rice varieties (long-grained rice, polished Round-grained rice, and black rice) and the common wheat variety were milled into different flours with the mixture design approach to clarify the effect of proteins, fat, microelement, and total dietary fiber (TDF) on the quality of steamed bread. Thus, this research aimed to investigate the effect of different rice flours on the nutritional characteristics and sensory evaluation of steamed bread.

## Materials and methods

### Materials

The common wheat (Wenmai 2504, Kato, Prosky) and three different rice varieties were obtained from the 2021 harvest in Henan, Hubei, and Heilongjiang provinces. Long-grained rice (LGR) variety (*Oryza sativa* L. subsp. *indica* Kato) was provided by Jingshan Hongda grain and oil industry Co., Ltd. (Jingmen, Hubei, China). The polished round-grained rice (PRGR) variety (*Oryza sativa* L. subsp. *japonica* Kato) was provided by Yuanyang Kangjian Co., Ltd. (Xinxiang, Henan, China). Black rice (BR) variety (*Oryza sativa* L. cv. Khao Niaw Dam Peuak Dam) was provided by Wuchang Changxinxing Food Co., Ltd. (Heilongjiang, China), and Wenmai 2504 (W 2504) as the common wheat was purchased at farmers’ markets (Jiaozuo, Henan, China).

### Sample preparation

The common wheat was manually cleaned and stored in a 4°C freezer. The incomplete wheat and other impurities were removed. The moisture content of wheat before milling was controlled at 15 ± 0.5% for 16 h. LFS-30 (Buhler, Wuxi, China) was used in milling of the common wheat, and assembling at 3B flour system. Whole wheat kernels were first used to remove the bran layer using a grain polisher (TYT200, Tianyang Machinery Co., Ltd., Shandong, China) before preparing the flour. The different rice flours were collected by the universal grinder. The mixed flour was prepared with LGR (A), polished round-grained rice (B), and black rice (C). According to the orthogonal test, the ratio of A: B: C was 1: 1: 1, 1: 2: 2, 1: 3: 3, 2: 1: 2, 2: 2: 3, 2: 3: 1, 3: 1: 3, 3: 2: 1, and 3: 3: 2, respectively. The dough was prepared using the method of Chen (41). After preparation, the dough was put in a 4°C refrigerator to cool. For each dough, samples were prepared in duplicate.

### Chemical analyses of the different rice flours

The lipid content of the different rice flours and wheat flour was determined using the method of Moreau with some modifications (42). Moisture was measured according to the National Standard of the People’s Republic of China GB 5009.3-2016. The protein content of the different flours was determined by the Kjeldahl method. In a 100 ml round bottom flask, 0.5 g of various rice and common wheat samples were mixed with 4 ml of concentrated sulfuric acid, and the mixture was heated to 440°C with a conventional convection-conduction heating system until boiling. However, the heating time should not exceed 3–5 min.

### Analysis of amino acids

Amino acids analysis of the rice and common wheat samples was accomplished according to the method of Du (43). The rice and common wheat samples (100 mg) were hydrolyzed with 10 ml 5 M NaOH at 110°C for 20 h. The hydrolyzate was transferred and dissolved with deionized water in a 50 ml volumetric flask. The mixture was then filtered through a 0.45 μm of nylon syringe filter (Filtrex Technology, Singapore). The analysis of amino acids from different rice and common wheat samples was determined with an automatic amino acid analyzer (Biochrom 30+, Cambridge, UK). Amino acids were derivatized with ninhydrin reagent for postcolumn (0–50 ml/h) and determined at wavelengths of 570 nm (for the quantitation of α-amino acids) and 440 nm (for the imino acids). Amino acids of different samples and standard solution were analyzed under the same conditions, and all the above measurements were performed in triplicate.

### Determination of total dietary fiber and microelement

The content of TDFs was determined according to the method of Prosky (44). Microelement determinations of the rice and common wheat samples were performed using an Agilent 240FS atomic absorption spectrometer (Agilent Technologies, Santa Clara, CA, USA) equipped with flame atomization [a mixture of acetylene (2.9 L/min) and air (13.5 L/min)] (45). The emission mode of K is set to 766.5 nm.

### Preparation of rice dough and steamed bread

Different ratios of rice flours into wheat flours were made to steamed bread according to the sponge dough method of He with some modification (46). The recipe of steamed bread is as follows: The common wheat flour is the base flour, and the different rice flours are added by 5, 10, 15, 20, 25, and 30% with the total amount being 100 g. Dry yeast, 1 g (Anqi, Anqi Yeast Co., Ltd., Hubei, China) was added into water (30°C). A total of 100 g samples were mixed with yeast/water which Farinograph water absorption rate up to 85% in a dough maker (B10, Henglian Food Machinery Co., Ltd., Guangdong, China). After mixing at medium speed for 10 min with the appropriate amount of warm water (38°C), and using a rolling pin to press the sheet 20 times, the dough was divided into three parts, and shaped by hand into a round, long, and straight dough with a smooth surface. Then, the dough pieces were proofed for 2 h at 30°C with 80% RH, and remixed flour and dough by hand until the dough was sticky. After that, the proofed steamed bread was put in a steamer (ASD, Zhejiang, China), when the water boiled, and steamed for 20 min.

### Sensory evaluation of steamed bread making

The numbers of panelists were 18 (10 men and 8 women). Panelists included 18 adults between the ages of 27 and 65. They were selected, screened, and recruited according to international standards (47). A total of 15 of them were university faculty, and 13 of them had received theoretical and practical training in sensory analysis and experience in tasting different flour products such as bread, steamed bread, or noodles. The other five had not participated in the panel discussion before. Assessors were not paid for participating in the panel.

### Statistical analysis

Data were collected for analysis of variance to determine differences between different rice flours and to investigate the effect of mixture design approach on farinograph and extensometer performance. Significance analysis was performed using Statistica 7.0 software to study the relationship between rheological properties and mixture design approach. Data were reported as the mean (standard deviation SD) of triplicates.

## Results and discussion

### Physicochemical properties of different-grained rice flours

The chemical composition of the different rice flours and wheat flour was shown in Table 1. The protein content of different rice flours was 27.24–43.04% lower than that of the common wheat. Protein is the main food component of humans and other animals. However, there are still challenges in formulating gluten-free products with rice because rice flour does not function as wheat gluten, which is known as an essential structural protein (48). The fat and total TDF content were almost lower than the common wheat. The fat of wheat is mainly concentrated in the germ; however, the fat of rice flour is mainly concentrated in the bran (49). TDF was mainly concentrated in the pericarp and seed coat, while the content of endosperm was very few. The content of fat was abundant in black rice flour because the rice bran was reserved in the process.

**TABLE 1:** Physicochemical index of different rice and common wheat.

Cultivars	Moisture content (%)	Protein (%)	TDF (%)	Fat (%)
W 2504	12.7 ± 0.207	10.9 ± 0.014*	1.20 ± 0.707*	1.5 ± 0.236
LGR	14.2 ± 0.216*	7.93 ± 0.007	0.72 ± 0.028	0.9 ± 0.038
PRGR	13.7 ± 0.124	7.70 ± 0.014	0.62 ± 0.071	0.6 ± 0.017
BR	11.6 ± 0.141	7.50 ± 0.134	0.81 ± 0.071	1.9 ± 0.070*

*Means within each wheat category followed by the same letter are not significantly different at *p* < 0.05, the water content is dry base content.

### Distribution of amino acid, total amino acid, and essential amino acid content

The amino acid contents for different rice flours and wheat flour were shown in Table 2. The nutritional characteristics of different rice flours mainly refer to the balance of protein content and amino acid composition, and the essential amino acid content is the key to determining the nutritional quality of different rice flours (50). TAA and EAA contents of different rice flours were lower than those of common wheat flour, and TAA content was 34.12–40.46% lower than that of common wheat flour. The essential amino acid content of different rice flours was 15.2–21.93% lower than that of common wheat. The results showed that the quantity and quality of protein and amino acids of common wheat flour were greater than those of different rice flours. Due to the amino acid composition characteristics of different rice flours, people can directly eat them to meet nutritional needs, and can also be used as breeding materials (51). The arginine content of different rice flours was higher than that of common wheat: Long-grain rice flour increased by 44.19%, polished round-grained rice by 21.74%, and black rice flour by 34.78%.

**TABLE 2:** Amino acid composition of different rice and common wheat (g/100 g).

AA	W 2504	LGR	PRGR	BR
Asp	0.5	0.67	0.68	0.65
Thr	0.31	0.29	0.28	0.28
Arg	0.43	0.62	0.68	0.56
Pro	1.32	0.33	0.32	0.3
Tyr	0.3	0.24	0.29	0.22
Ala	0.37	0.44	0.42	0.42
Val	0.45	0.46	0.46	0.44
His	0.21	0.2	0.2	0.19
Met	0.15	0.13	0.13	0.1
Ile	0.38	0.32	0.3	0.28
Cys	0.16	0.11	0.12	0.11
Phe	0.48	0.45	0.43	0.38
Ser	0.58	0.44	0.44	0.39
Glu	4.26	1.5	1.48	1.26
Gly	0.44	0.32	0.33	0.33
Lys	0.23	0.28	0.27	0.31
Leu	0.8	0.62	0.62	0.55
TAA (g/100g)	11.37	7.42	7.45	6.77
EAA (g/100g)	3.42	2.9	2.9	2.67
EAAI	91.64	78.86	78.27	73.38

*Tryptophan content is less, no determination results.

**TAA, total amino acid content; EAA, essential amino-acid content; EAAI, essential amino acid index.

### Determination of the microelement in different rice flours

The microelement contents for different rice flours and wheat flour were shown in Table 3, and the content of zinc in black rice flour was 132.32% higher than that of W 2504. Zinc has a role in helping intellectual growth and development (52). The content of sodium in black rice flour was 160.81% higher than that of W 2504. Sodium is an essential trace element for the human body, an important part of water balance, and a complex component of pancreatic juice, bile, sweat, and tears. Therefore, sodium can promote physical development and increase the body’s ability to resist disease. At the same time, sodium can also regulate tissue respiration and prevents fatigue (53). However, the content of other microelements in different rice flours was lower than that of W 2504. Therefore, the mixture design approach can improve their nutritional value.

**TABLE 3:** Microelement content of different rice and common wheat.

Cultivars	Zn^++^	Fe^++^	Mg^++^	Na^+^	Ca^++^
W 2504	16.4 ± 0.071	35.2 ± 1.13*	320 ± 2.828*	27.3 ± 1.414	270 ± 1.414*
LGR	14.7 ± 0.141	16.2 ± 0.283	280 ± 2.828	17.1 ± 0.141	120 ± 4.243
PRGR	11.2 ± 0.028	16.4 ± 0.057	158 ± 2.828	19.3 ± 0.141	191 ± 1.414
BR	38.1 ± 0.283*	16.2 ± 0.156	147 ± 1.414	71.2 ± 1.414*	120 ± 2.828

*Means within each rice category followed by the same letter are not significantly different at *p* < 0.05.

### Effect of mixture design approach on nutritional characteristics

Flour products are a major part of the Chinese daily diet, with wheat as the main source, but are mainly made from the seeds of common wheat. However, different rice flours contain most of the valuable protein, micronutrients, and phytochemicals found in grains, and if included in flour or used as food ingredients, they would go to a long way toward improving the nutritional quality of human food (54). Nutritional characteristics of mixed flours from different rice flours and wheat flour were shown in Table 4. The results showed that the highest protein content was no. 8 (A_2_B_3_C_1_), 6 (A_2_B_3_C_1_), and 9 (A_3_B_3_C_2_). However, the highest value of EAAI was no. 8 (A_3_B_2_C_1_) and 6 (A_2_B_3_C_1_). The microelement content highest is the no. 3 (A_1_B_3_C_3_) and 6 (A_2_B_3_C_1_). Therefore, when paired with these factors, we choose no. 6 (A_2_B_3_C_1_) as a nutritional content more rich.

**TABLE 4:** Nutritional characteristics of different rice flour with different proportions.

	Combination	Protein (%)	TDF (%)	EAAI	Microelement (mg/kg)
1	(A_1_B_1_C_1_)	7.71 ± 0.028	0.70 ± 0.014*	76.84	181.09 ± 1.541
2	(A_1_B_2_C_2_)	7.67 ± 0.028	0.70 ± 0.014*	76.43	187.17 ± 3.068*
3	(A_1_B_3_C_3_)	7.65 ± 0.042	0.70 ± 0.014*	76.26	189.77 ± 2.503*
4	(A_2_B_1_C_2_)	7.71 ± 0.014	0.72 ± 0.014*	76.55	173.59 ± 2.248
5	(A_2_B_2_C_3_)	7.68 ± 0.014	0.72 ± 0.014*	76.34	180.08 ± 1.301
6	(A_2_B_3_C_1_)	7.74 ± 0.049*	0.67 ± 0.028	77.65	188.51 ± 2.135*
7	(A_3_B_1_C_3_)	7.71 ± 0.028	0.73 ± 0.028*	76.43	170.38 ± 2.291
8	(A_3_B_2_C_1_)	7.78 ± 0.042*	0.69 ± 0.014*	77.75	177.21 ± 1.117
9	(A_3_B_3_C_2)_	7.74 ± 0.028*	0.69 ± 0.028*	77.27	181.98 ± 2.80

*Means within each rice category followed by the same letter are not significantly different at *p* < 0.05.

### Effect of mixed flours on sensory analysis of steamed bread

**FIGURE 1:**
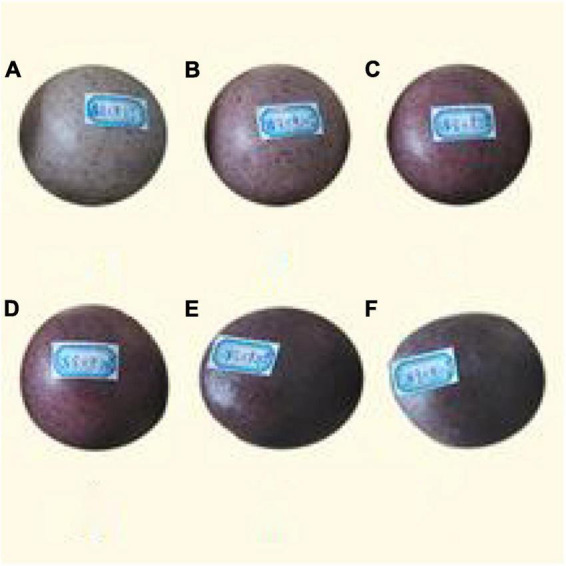
Images of steamed bread made by different rice flours [the added amount of panels **(A–F)**: 5, 10, 15, 20, 25, and 30%].

**FIGURE 2:**
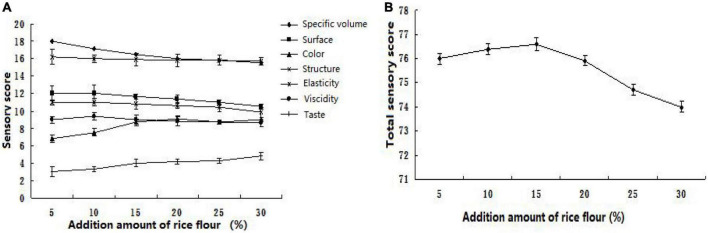
Sensory analysis of different rice flour with different proportions (**A:** Sensory score; **B:** Total sensory score).

As shown in Figures 1, 2, with the increase in the amount of different rice flours, the specific volume of steamed bread is getting smaller and smaller. Because different rice flours do not contain gluten, the steamed flours cannot form a gluten network structure. The results have shown that the different rice flours have some effects on weakened gluten network structure. The amount of different rice flours exceeded the ultimate value, so small size, low specific volume, bouncy toughness and bite, and poor shape would appear in the steamed bread. When the added amount of different rice flours exceeds 15%, the steamed bread gradually reduces elasticity and has a smaller specific volume.

As shown in Figure 2A, the specific volume of steamed bread is becoming smaller and smaller with the increase of colored rice composite powder. This is because rice does not contain gluten, which cannot form a gluten network structure. The research shows that the medium gluten flour is more suitable for making steamed bread, while the coarse grain flour has a certain role in weakening the gluten strength. Excessive addition will lead to low gluten strength, making steamed bread small in volume, low in specific volume, poor in elasticity and bite strength, poor in shape, and large and uneven in pulp structure (1, 3). So the appearance, structure, elasticity, and toughness are slightly reduced. The evaluation value of color and smell has been rising due to the special color and aroma of rice. As shown in Figure 2B, with the increase of colored rice composite powder, the total sensory evaluation score of steamed bread increased first and then decreased. When the added amount exceeds 15%, the elasticity of steamed bread gradually decreases, the taste becomes worse, and the specific volume becomes smaller. When the addition amount was 15%, the total score of colored rice steamed bread was the highest. As mentioned in the color section above, the reduced whiteness of the crust and crumbs can easily be attributed to the color in the rice flour. Color change of cereals may appeal to consumers (40).

## Conclusion

The effects of additions of different rice flours with different proportions on the nutritional characteristics and quality of steamed bread were investigated. Based on the analysis of protein, TDF, fat, amino acid composition, microelement, physicochemical index, nutritional characteristics, and quality of steamed bread, this study demonstrated that additions of different rice flours with different proportions primarily affected physicochemical index, nutritional characteristics, and quality of steamed bread. When the addition amount was 15%, the total score of colored rice steamed bread was the highest. The results of the current study offer opportunities for the steamed bread and noodle industry to use different rice flours as an ingredient for enhancing the nutrient-value amount of dough. The acceptability of dough with different proportions of different rice flours needs further study. The different rice flours have the potential to be utilized as nutrient-value foods.

## Data availability statement

The original contributions presented in this study are included in the article/supplementary material, further inquiries can be directed to the corresponding author.

## Author contributions

ST: data curation (equal), formal analysis (equal), investigation (equal), writing—original draft and review (equal), and editing (equal). YW: conceptualization (equal) and writing—review and editing (equal). ZC: formal analysis (equal), methodology (equal), writing—original draft (supporting), funding acquisition (equal), and writing—review and editing (equal). All authors contributed to the article and approved the submitted version.
